# Designing a cardiac surgery mortality risk model with spanish population

**DOI:** 10.1186/2197-425X-3-S1-A837

**Published:** 2015-10-01

**Authors:** M Pérez Cheng, P Pabón Osuna, JM González Santos, A Rodríguez Encinas

**Affiliations:** Intensive Care Department, University Hospital of Salamanca, Salamanca, Spain; Cardiology Department, University Hospital of Salamanca, Salamanca, Spain; Cardiac Surgery Department, University Hospital of Salamanca, Salamanca, Spain

## Introduction

The EuroSCORE II was designed with the purpose of improving the calibration of the previous additive and logistic version. This improvement has not been observed in our patients due to a global underestimation of mortality.

## Objectives

This study is aimed to design a cardiac surgery risk score to evaluate operative mortality in our patients. This is defined as in-hospital mortality or mortality by 30 days after the operation for patients discharged from the hospital.

## Methods

Data for all patients undergone heart surgery at the University Hospital of Salamanca, Spain, between 2001 and 2014 were collected. The data set was divided into a developmental subset for logistic regression modelling and a validation subset for model testing. Our results were compared with EuroSCORE II. Accuracy was defined with a receiver-operating characteristics analysis and calibration with a Hosmer-Lemeshow test. Data was analyzed with SPSS v.22.

## Results

In the developmental subset we included 3316 patients who had undergone cardiac surgery between 2001 and 2010, with 6.5% mortality. We identified predictors of mortality with *x*^*2*^ and t-Student tests (*p* < 0.05) and designed the model using forward logistic regression based on likelihood ratio statistics. We created a simplified clinical risk assessment tool derived from the logistic regression equation which included female sex, age (> 70 years), extracardiac arteriopathy, serum creatinine greater than 2 mg/dl, previous cardiac surgery, NYHA class IV, left ventricle ejection fraction lower than 30%, pulmonary artery pressure greater than 55 mmHg, critical preoperative state, urgent o emergency operation, isolated CABG and three or more procedures. The validation series included 1302 patients between 2011 and 2014. Discrimination assessed with the ROC curve was good with our model (AUC 0.76) and also with EuroSCORE II (AUC 0.75), though it was poor calibrated in the validation data (actual mortality: 7.2%, predicted: 5.6%). Good calibration was found with our model (predicted mortality: 8.3%) with a non significant Hosmer-Lemeshow test (*x*^*2*^ 6.14, *p*: 0.52).

## Conclusions

Differences in our population explain the poor calibration we found with EuroSCORE II: older and sicker patients, more women and less isolated CABG are some of these differences. Any existing model should be tested to assess calibration in a specific population. Models derived from local subjects ensure a better performance if the goal is risk prediction.
